# Construction of public health core competence and the improvement of its legal guarantee in China

**DOI:** 10.3389/fpubh.2023.1125591

**Published:** 2023-02-20

**Authors:** Lansong Huang, Qinglin Yu, Quansheng Wang

**Affiliations:** Law School, Shandong University, Weihai, China

**Keywords:** public health core capacity, International Health Regulations (IHR), public health events, legal safeguard capacity, public health

## Abstract

Public health core capacity, first established by the 58th United Nations General Assembly in 2003 and recognized by the World Health Organization when “the International Health Regulations” were revised, refers to the basic and necessary capacity to allocate human, financial, and material resources for the prevention and control of public health events that a country or region should have. It includes national and regional levels, and its constituent elements and their basic requirements differ, but public health core capacity building at both national and regional levels requires certain legal safeguards. At present, there are still some problems, including the imperfect legal system, conflicting legal norms, the non-sufficient supply of local legislation, and the weak operability of legislation in the legal guarantee of public health core capacity building in China. China should make improvements in terms of comprehensive cleaning of existing public health laws, strengthening their post-legislative evaluation, adopting parcel legislation, strengthening legislation in key areas of public health, and promoting the supply of local legislation. The goal is to provide a perfect and comprehensive legal system to guarantee the construction of China's core capacity in public health.

## 1. Introduction

In 2020, the COVID-19 pandemic was continuously declared a “public health emergency of international concern” by the World Health Organization, and up to this day, this public health emergency has brought immeasurable injuries and property damage to countries around the world. The impact is still further expanding. The World Health Organization called on countries to respond proactively to avoid further damage. In a public health emergency, different countries have different political positions, different attitudes toward public health emergencies, different emergency response capabilities, and different levels of damage. Public health emergencies are severe tests of a country's emergency capacity building. Of course, they also give some inspiration to countries around the world on how to further improve their core capacity for public health emergencies, which is an important measure to prevent public health emergencies.

## 2. The proposal for core capacity building in public health

The concept of “public health capacity” as an international rule was first introduced on October 22, 2003, when the 58th General Assembly of the United Nations adopted the “United Nations Draft Resolution on Enhancing Capacity Building in Global Public Health,” in which it was proposed that to meet the new challenges of public health, all Member States must strengthen public health capacity building, and the Member States were urged to revise the “International Health Regulations” as a high priority to enhance global public health capacity building.

The World Health Organization developed “The International Health Regulations (IHR),” an international health law. The earliest name was the “International Public Health Regulations,” adopted by the Fourth World Health Assembly in 1951, and the 22nd World Health Assembly in 1969 revised and enriched the IHR and changed its name to the “International Health Regulations,” and then it went through revisions in 1973 and 1981. The current text is called the International Health Regulations (1), which is the revised text adopted by the 58th World Health Assembly on May 23, 2005, and entered into force on June 15, 2007.

The 2005 revision was in response to the “United Nations Draft Resolution on Enhancing Capacity Building in Global Public Health,” adopted by the 58th United Nations General Assembly in 2003. This revision included many contents, including cooperation with international organizations in Article 14 of the Regulation; the decision process in Article 54; the notification process in Article 65; and the revision of public health capacity building for each state party: first, to build, strengthen, and maintain the public health capacity established in the Regulation and to mobilize the resources for this purpose; second, to work with the international organizations in Article 14 of the Regulation; and third, to work with the international organizations in Article 65 (2).

International Health Regulations (1) include nine annexes and two appendices. In Supplementary material, two core competencies are mentioned: first, a core competency for surveillance and response; and second, a core competency for designating airports, ports, and land crossings. However, in Article 1 of the regulation, the term “definition” does not define “public health core capacity.” What are the core competencies of public health, and what is the difference between public health core competencies and public health competencies? What are the changes that have taken place between the “public health core competencies” proposed by the 58th UN General Assembly and the “public health core competencies” established by the International Health Regulations (1) as revised by the 58th WHO General Assembly? However, the International Health Regulations (1), as revised by the World Health Assembly in 2005, established the concept of “core public health capacity” and required state parties to build their “core public health capacity.”

We searched the relevant databases and found 20 papers with the title of “Public Health Core Competencies,” 5 papers in “web of science,” 1 paper in “ScienceDirect,” 6 papers in “Wiley Online Library” and 1 paper in “Pubmed.” We searched for 5 papers on “web of science,” 1 paper on “ScienceDirect,” 6 papers on “Wiley Online Library,” and 8 papers on “Pubmed”After careful reading, only two papers were specifically related to the “public health core competencies” in the International Health Regulations, one of which examined the “public health core competencies.” One paper examined the risk identification competencies in the “Public Health Core Competencies” (3); and one paper combed through the list of European public health core competencies (4).

## 3. Structural elements of public health core capacity building

Although the International Health Regulations (1) does not define “public health capacity” or “public health core capacity” in its definition clause of Article 1, it defines “public health core capacity” in Supplementary material. First, public health surveillance, reporting, notification, verification, response, and cooperation capabilities are required from the grassroots level (community public health) to the mid-level public health level and the national level. Of course, the core public health capabilities at different levels are different; second, the core public health capabilities are required at designated airports, ports, and land crossings during normal times and in the event of “public health emergencies of international concern.”

“Public health capacity” and “core public health capacity” are two distinct and related concepts, which were introduced by the 58th United Nations General Assembly in 2003 and are based on: first, the need for Member States to work to halt and reverse the spread of AIDS and the incidence of other major diseases such as malaria by 2015; second, to recognize that globalized trade and increasing international travel can increase the risk of international transmission of infectious diseases and pose new challenges to the public health of countries; and third, a focus on the harmful effects of infectious diseases in human beings, and the heavy burden of disease in the poor, especially in developing countries. The United Nations General Assembly has called on the Member States to strengthen “public health capacity” as a critical component of promoting economic and social development (5). Although the “public health capacity” proposed by the United Nations General Assembly served as a reference for the World Health Organization to improve the relevant international regulations, it was also the motivation for the World Health Organization to revise the International Health Regulations, which were revised by the WHO General Assembly in 2005 and renamed the International Health Regulations (1). Although Supplementary material of the International Health Regulations (1) establishes the “public health core capacities” and “public health core capacities” of ports at the national level, it is no clear definition of “public health core capacity” in the regulation. From the “public health capacity” established by the United Nations and the “public health core capacity” established by the International Health Regulations (1), the first component is the ability to prevent the spread of infectious diseases at the level of national sovereignty; second, at the level of state-to-state relations, it is important to cooperate to prevent both the international spread of infectious diseases and the impact of international spread of infectious diseases on international economic trade. Although these two terms differ by two words, they have the same substantive meaning in terms of the UN General Assembly resolution and the Carlson et al. (6) and International Health Regulations (1).

The International Health Regulations (1) on the core competency elements of public health is concentrated in Tables A, B in Supplementary material. Table A is “Core capacity requirements for surveillance and response,” and Table B is “Core capacity requirements for designated airports and ground crossing. Table B provides two types of capabilities for airports, ports, and land transportation, namely “At all times” and “For responding to events that may make up a public health emergency of international concern.” In this paper, the authors do not distinguish between “At all times” and “For responding to events that may constitute a public health emergency of international concern.”

The International Health Regulations (1) (Supplementary material) on the basic elements of the national “public health core capacity” include the following.

### 3.1. First, having relevant institutional measures

For example, the system of public health emergency response plans, the system of public health-related laws and regulations, the national emergency response system, etc. The country should have a system of public health-related laws and regulations and have an effective implementation method (7).

### 3.2. Second, having appropriate institutions and personnel

Such as public health emergency command agencies, grassroots health organizations, health management agencies, administrative coordination agencies, professional health personnel, professional laboratory testers, health managers, coordinators, etc. It means a country should have a sound public health management system, a public health agency system, public health command, and coordination agencies, public health testing and inspection agencies, and relevant professional personnel, such as doctors, nurses, testers, etc. (8).

### 3.3. Third, having public health-related equipment and facilities

Such as laboratories with testing capabilities, medical protection equipment, logistics facilities, medical diagnostic facilities, port of entry-related public health facilities, isolation facilities, and so on, is required by “International Health Regulations (1),” the core capacity of public health cannot be improved without the improvement of these facilities and equipment (9).

### 3.4. Fourthly, having generous material and financial support

Although the corresponding institutions and facilities also need a certain amount of financial support, besides financial support for public health, it is necessary to have a series of public health material security backups. Without a solid backup, especially in the event of public health emergencies of international concern, it will be caught unprepared and maybe even abandon the previous work. The International Health Regulations (1) stipulate that besides the support provided by the World Health Organization to developing countries, each State Party shall, within its capacity, provide public health support to developing countries and third-world countries, which mainly includes, material and financial support besides technical public health support (10).

## 4. Legal protection status and problems of public health core capacity building in China

China is a contracting member of the International Health Regulations (1). In the statement submitted by the Chinese government to the World Health Organization, the Chinese government pledged to strengthen its core public health capacity building, “developing technical specifications for surveillance, reporting, assessment, determination, and notification of public health emergencies of international concern,” and “establishing a cross-sectoral information exchange and coordination mechanism for implementing the Regulations” (1).

Article 14 of the International Health Regulations (1) specifies that each State Party must, no later than 5 years after entry into force of the regulations, “develop, strengthen, and maintain the capacity to respond rapidly and effectively to public health risks and public health emergencies of international concern.” According to the International Health Regulations (1), the revised regulations have been effective since June 15, 2007. In 2013, the General Office of the State Council forwarded to the Health and Family Planning Commission and eight other departments' the “Guidance on Effective Implementation of the International Health Regulations (1) Accelerating Core Capacity Building for Public Health Emergencies,” proposing to establish and improve the public health capacity-building measures. Some scholars have conducted a systematic examination of public health capacity building in China. Using a stratified sampling method, the authors selected 7 provinces, 64 prefecture-level cities, and 140 counties (cities and districts) in China and analyzed the construction of their surveillance, response, risk communication, preparedness, laboratory capacity, infection control, and material and financial support capacity required by the International Health Regulations (1) about public health emergency core capacity, and concluded that the International Health Regulations (1) public health emergency core capacity indicators apply in China; the International Health Regulations (1) public health emergency core capacity indicators lack institutional construction; and the three levels of provincial, municipal, and county (city and district) show a decreasing trend in public health emergency core capacity (11). Regarding the achievements of public health core capacity building at China's airports, ports, and land crossings, some information shows that “in 2014, all 259 open ports to the outside world in operation met all core capacity standards” (12). However, there are also some problems, such as the overall level of capacity building needs to be improved; laboratory capacity has not yet reached the International Health Regulations (1) requirements, and the ability to deal with emergencies also needs to be improved.

As mentioned earlier, sound public health institution building is an important indicator of a country's core public health capacity, and, as mentioned earlier, it is also the institutional guarantee for the other three elements of core public health capacity. The legal system is at the core of these systems. After over 70 years of development, China has formed a relatively complete public health legal system, which has now formed four levels: public health laws; administrative regulations, local regulations, rules, and corresponding public health standards. “Food Safety Law,” “Agricultural Product Quality and Safety Law,” “Product Quality Law,” “Prevention and Control of Infectious Diseases Law,” “Medical Practitioner Law,” “Vaccine Management Law,” “Emergency Response Law,” “Water Pollution Prevention and Control Law,” “Solid Waste Pollution of the Environment Prevention and Control Law,” “Animal and Plant Protection Law,” “Chinese Medicine Law,” “Basic Medical Sanitation and Health Promotion Law,” “Biosecurity Law,” and other laws. Some scholars have summarized the achievements and characteristics of China's public health legal system. First, the form of law has initially built a relatively complete legal framework system for public health epidemic prevention and control. Second, while it focused on the content of the regulation, the relevance, and operability of the legal system for epidemic prevention and control have been significantly improved. Third, the legal system has achieved a systematic presentation for strengthening the prevention and control of major public health epidemics (13).

The COVID-19 pandemic has continuously been declared a “public health emergency of international concern” by the World Health Organization, which is a severe test of the core public health capacity of countries around the world, as well as a test of the legal protection system for building core public health capacity in China. Although China's central and local legislatures have done a lot of work, enacted and amended many laws related to public health, and improved the legal protection capacity of China's public health core capacity building, the following problems and shortcomings still exist in China's public health legal regulation:

### 4.1. First, the system was not sound

The soundness of a country's public health legal system depends on whether it comprehensively regulates the objects and scope of public health change, and it should have a reasonable structure. China now has many public health laws, but another is a lack of basic public health laws to unify the public health legal system. China now has many public health laws, but laws on the objects and scope of public health adjustments, such as public health event prevention, public health emergency response, and so on, have yet to be introduced.

### 4.2. Second, the conflict of laws undermines the authority and operability of the law

China's current public health laws are still relatively large, but because it enacted these laws in different periods, some provisions and systems have a certain conflict, such as the provisions about early warning information. “Prevention and Control of Infectious Diseases Law” provide that it limits the subject of early warning of infectious diseases to the State Council's administrative department of health and the people's governments of provinces, autonomous regions, and municipalities directly under the Central Government. While the “Emergency Response Law” specifies that the subject of early warning information is the government above the county level; there is also a conflict with provisions of the emergency requisition system. Article 12 of the “Emergency Response Law” provides that the relevant people's government and its departments may requisition the property of units and individuals in response to an emergency. Article 45 of the “Prevention and Control of Infectious Diseases Law” provides that the State Council has the right within nationwide or across provinces, autonomous regions, and municipalities directly under the Central Government, and local people's governments above the county level have the right within their administrative regions to urgently mobilize personnel or call for reserve materials, and temporarily requisition housing, transportation, and related facilities and equipment. The conflicts and contradictions in our current public health laws not only affect the authority of our laws, but they undermine the operability of the laws.

### 4.3. Third, the supply of local public health legislation is insufficient

China's public health core capacity building, provincial, municipal, and county public health core capacity building, shows a decreasing trend. The more to the grassroots, the core capacity building is insufficient, and some serious infectious diseases usually occur at the grassroots. If grassroots prevention is not effective, the trend will expand. International Health Regulations (1) require public health core capacity building in sovereign countries to be divided into three levels: the local community level and/or primary public health response level; the intermediate public health response levels, and the national level, the first two levels of public health core capacity building are issued in China within the scope of local public health core capacity building. The main reason for the lack of local public health core capacity building should be the lack of local public health legislative protection capacity, which is prominently expressed in the lack of local public health legislative supply. China implements a two-tier legislative system at the central and local levels. The local legislature has two tasks: one is implementing legislation, the central legislation of the relevant provisions is needed to be further specified in the local, to be practicable and to implement the higher law; the other is the creation of legislation, the local according to the actual local situation, in the absence of the higher law, to make institutional provisions. In China's current local public health legislation, first, there is a lack of local implementation of the central public health legislation, so the implementation and enforcement are not in place; second, lack of local creative legislation for the local public health condition. As a result, the supply of local public health legislation is insufficient, and the rule of law for public health lacks effective institutional support and protection.

### 4.4. Fourthly, the operability of our public health legislation is insufficient

There are conflicts in public health legislation, thus making the legislation difficult to implement and not operable. An example is the “Prevention and Control of Infectious Diseases Law.” The law mainly applies to statutory infectious diseases. For new and sudden outbreaks of infectious diseases, only when it is necessary to take preventive and control measures for Class A infectious diseases can the health administrative department of the State Council report to the State Council for approval and then be announced and implemented. The “Prevention and Control of Infectious Diseases Law” and the “Emergency Response Law” on the restriction of personal freedom are also vague, as the provisions on the subject, authority, content, and procedures for taking administrative coercive measures are unclear enough, and the administrative discretion is too large, which may easily lead to disputes (14). Of course, other public health laws and regulations also exist with some vague provisions and a lack of operability.

## 5. Suggestions for improving legal safeguards for building core public health capacity in China

### 5.1. Thoroughly clean up public health laws and regulations

The COVID-19 pandemic exposes deficiencies and shortcomings in China's public health laws and regulations. In April 2020, the Legal Affairs Committee of the Standing Committee of the National People's Congress (NPCSC) submitted a research report to the NPCSC, “Report of the Legal Affairs Committee of the Standing Committee of the National People's Congress: Strengthening Public Health Legislation and Amending the Law Must Grasp Five Important Principles Requirements (15).” The report points out that “the epidemic has revealed that the problems in legislation are mainly: there are still gaps, weaknesses, and shortcomings in the law, and some legal provisions are not sufficiently connected or even conflict; there is a real need for systematic revision and improvement.” Therefore, the legislative bodies at all levels in China should, within their legislative authority, conduct a comprehensive cleanup of their respective public health laws and regulations and plan a perfect legislative amendment plan for public health laws, just in time to take this opportunity to establish and improve China's public health legal system.

### 5.2. Strengthen the post-legislative evaluation study of public health laws

Post-legislative evaluation is the evaluation of the implementation effect of the law by the relevant subjects. It analyzes the relationship between the implementation effect of the law and the legal system and puts forward suggestions for the creation, reform, and abolition of the legal system in response to the legal norms (16). China's public health laws have been enacted over a long period, and some of them have failed to meet reality, exposing their problems. The relevant legislative bodies in China should promptly start a systematic or individualized assessment of the enacted public health laws, to provide a mechanism for legitimizing the improvement of public health laws.

### 5.3. Implement a parcel-type legislative model to realize the harmonization of the legal system

The conflict of law not only affects the authority of the law, but it hinders its effective implementation and realization. Although the number of public health law departments in China is large, although there are differences in objects and contents, there are inevitable overlaps. To maintain the consistency and coordination of the legal system and to safeguard the principle of “unity of the legal system” established by the Constitution, we should adopt a package legislative model. The so-called wrapped legislative model is the “package amendment.” “When the legislature considers a bill, to achieve a legislative purpose, the relevant provisions originally scattered in individual laws are put together in a single law, a one-time revision or update of the legislative approach (17).” According to Luo Chuanxian, the adoption of the parcel legislation model can save legislative time, avoid contradictions and conflicts between laws, and guarantee the organic combination of laws and legal provisions, etc. ^[11]^ According to Chen Xinmin, the advantages of the parcel legislation model are 1) to enhance the efficiency of legislation; 2) to improve the integrity of the legal rules; and 3) to avoid the negligence of legislators (18). The adoption of a wraparound legislative model for the revision of China's public health laws can effectively prevent the conflict of legal norms, promote the harmonization of the legal system, and enhance the operability of public health laws.

### 5.4. Strengthen legislation in key areas of public health in China

The COVID-19 pandemic has also shown the shortcomings and deficiencies of public health laws in China, and we need to timely strengthen legislation in key areas of public health to make up for this legislative shortcoming and build a sound public health legal system. Some scholars have proposed that China should enact an “Emergency Law” based on the “Emergency Response Law” and amend the “Wildlife Protection Law” as soon as possible, which is the current legislation in key areas of public health in China (19). The Standing Committee of the National People's Congress adjusted its annual legislative plan on June 1, 2020, adjusting the legislation in key areas of public health to “amend the Animal Epidemic Prevention Law, the Wildlife Protection Law, Frontier Health and Quarantine Law, Infectious Disease Prevention Law, Emergency Handling Law, etc. (20).” Accelerating legislation in key areas of public health compensates for legislative shortcomings, and builds a solid public health legal system that serves as the institutional foundation and reliance for legal protection of public health core capacity building.

### 5.5. Increase the availability of local public health legislation

China has implemented a two-tier legislative system at the central and local levels, and the local legislatures must play their position and role in local governance, use their legislative power well, strengthen their local public health legislation effectively, and focus on the adaptability and operability of their local public health legislation. For example, Hubei developed the “Provincial Public Health Safety Emergency Management Regulations” into the annual legislative plan; Jiangsu Province brought the “Jiangsu Province Implement People's Republic of China Infectious Disease Prevention and Control Law approach (revised)” and “Jiangsu Province Emergency Response Regulations” into the annual legislative plan; Anhui Provincial People's Congress Standing Committee put “Anhui Province Infectious Disease Prevention and Control Regulations” into the annual legislative project; Yunnan Province took the “Measures for implementing the People's Republic of China Infectious Disease Prevention and Control Law” into the 2022 legislative project; the Standing Committee of Chongqing Municipal People's Congress incorporated “Emergency Regulations on Public Health Emergencies” into legislative projects (21). In terms of the actual implementation effect, in 2022, local legislatures in China have effectively strengthened local legislation on public health, issued 23 decisions on epidemic prevention and control, introduced 16 pieces of local legislation on wildlife protection (including amendments), developed 4 pieces of legislation on the emergency management of public health events, and published 10 pieces of legislation on Chinese medicine, which have promoted the supply of local public health legislation and have improved the legal protection capacity of local public health core capacity building to an extent.

## Data availability statement

The original contributions presented in the study are included in the article/Supplementary material, further inquiries can be directed to the corresponding author.

## Author contributions

QW determined the basic framework of the thesis, clarified the basic ideas and central thoughts of the paper, and revised and reviewed the thesis. LH and QY collected and organized the literature, wrote the first draft of the thesis, made the preliminary layout of the thesis, unified, and standardized the format of the literature, etc. All authors contributed to the article and approved the submitted version.
