# Supplementary figures and images for: Construction of public health core competence and the improvement of its legal guarantee in China
Source: Front Public Health. 2023 Feb 20;11:1125591. doi: 10.3389/fpubh.2023.1125591 (PMC9986289; doi:10.3389/fpubh.2023.1125591)

**Annex 1 to the International Health Regulations**


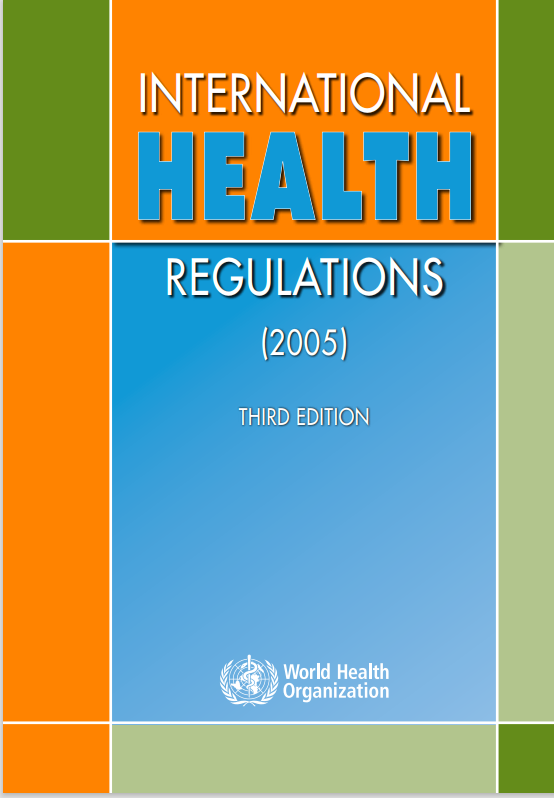


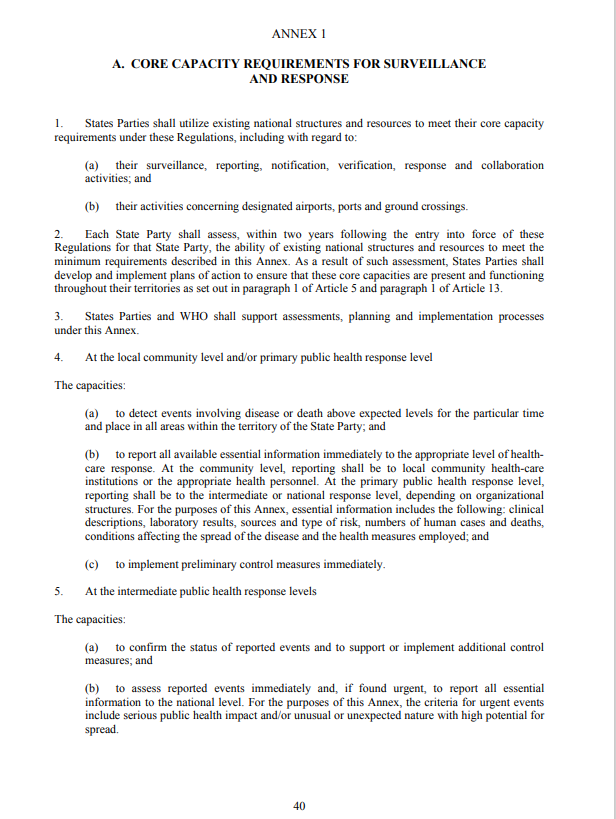


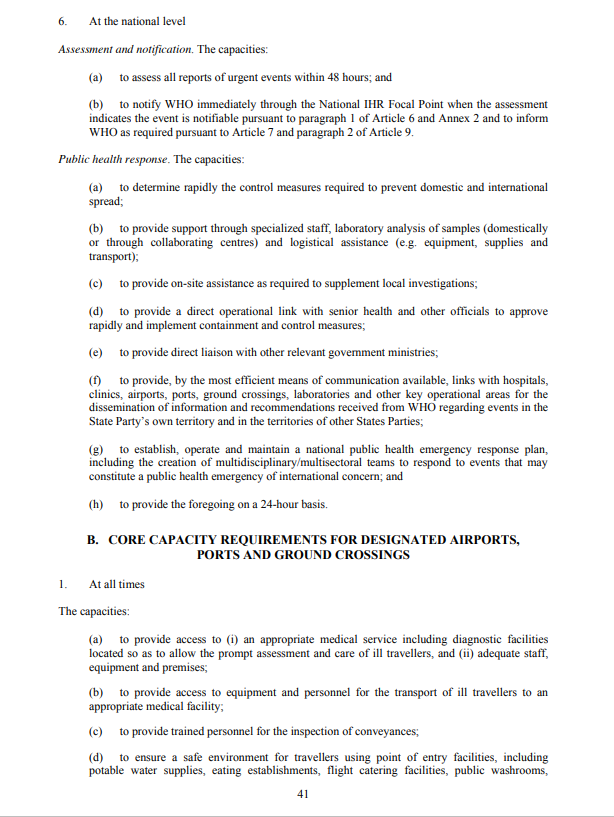

Supplement: Supplementary file 1 [file Data_Sheet_1.docx]
